# Optimizing Disaster Preparedness Planning for Minority Older Adults: One Size Does Not Fit All

**DOI:** 10.3390/ijerph20010401

**Published:** 2022-12-27

**Authors:** Omolola E. Adepoju, Luz Herrera, Minji Chae, Daikwon Han

**Affiliations:** 1 Department of Health Systems and Population Health Sciences, Tilman J. Fertitta Family College of Medicine, University of Houston, Houston, TX 77204, USA; 2 School of Law, Texas A&M University, Fort Worth, TX 76102, USA; 3 Humana Integrated Health System Sciences Institute, University of Houston, Houston, TX 77004, USA; 4 Department of Epidemiology and Biostatistics, Texas A&M University School of Public Health, College Station, TX 77843, USA

**Keywords:** minority, older adults, disaster preparedness

## Abstract

By 2050, one in five Americans will be 65 years and older. The growing proportion of older adults in the U.S. population has implications for many aspects of health including disaster preparedness. This study assessed correlates of disaster preparedness among community-dwelling minority older adults and explored unique differences for African American and Hispanic older adults. An electronic survey was disseminated to older minority adults 55+, between November 2020 and January 2021 (*n* = 522). An empirical framework was used to contextualize 12 disaster-related activities into survival an0000000d planning actions. Multivariate logistic regression models were stratified by race/ethnicity to examine the correlates of survival and planning actions in African American and Hispanic older adults, separately. We found that approximately 6 in 10 older minority adults did not perceive themselves to be disaster prepared. Medicare coverage was positively associated with survival and planning actions. Income level and prior experience with disaster were related to survival actions in the African American population. In conclusion, recognizing the gaps in disaster-preparedness in elderly minority communities can inform culturally sensitive interventions to improve disaster preparedness and recovery.

## 1. Introduction

With an increasing frequency and severity of natural hazards caused by anthropogenic climate change, countries with aging populations face growing challenges in caring for those in advanced age [1]. While historically, disaster risk management has focused on response to disasters, current disaster risk management endeavors include risk prevention and mitigation and disaster response and recovery measures [2]. Certain groups, such as minorities and older populations, are more vulnerable to suffer from the consequences of disasters [3,4,5]. Despite having the greatest risk, studies show that minority older adults tend to be underprepared and are not able to fully respond and recover from disasters or pandemics [4,6]. Reasons for this include not having the monetary resources to purchase items necessary to prepare for disasters, such as an emergency supply of food, water, medications, and a battery-powered radio or television [7,8]. More so, many are unaware of community resources available to them, resulting in the decision to shelter in place instead of evacuating [9]. Factors contributing to their inability or reluctance to leaving dangerous environments include limited transportation, physical impairments, lack of communication, and the lack of financial means [6,10].

Minority older adults may also be culturally reluctant to follow advice from authorities due to mistrust of the government or having a low perception of threat [10]. Prior experience may contribute to or hinder disaster preparedness. Individuals who suffered greatly in past disasters know how difficult and slow it is to obtain emergency aid relief [7]. As a result, they tend to prepare better for disasters but remain underprepared nonetheless. On the other hand, individuals who mildly suffered from disasters and survived without great implications will generally underprepare for future disasters, therefore making them at high risk for morbidity and mortality [11]. Furthermore, minority older adults may face cultural and linguistic barriers that hinder their ability to prepare for disasters. Individuals that primarily speak another language (other than English) may rely on other sources for information. Translated messages and announcements may lose important contextual and culturally relevant information. Such communications can ultimately be confusing and disregarded by minority older adults [12].

Although the adverse impacts of disasters are preventable—to a degree—with appropriate planning and resources, the interruption of medical care and access to medications have long been considered major causes of increased mortality rates following major disasters. In an attempt to prevent disaster-related fatalities, the Centers for Medicare and Medicaid (CMS) issued the Emergency Preparedness Requirements for Medicare and Medicaid Participating Providers and Suppliers. This new rule established national requirements for Medicaid and Medicare providers so that measures to plan and coordinate with emergency preparedness systems were taken prior to disasters occurring. Similar bills were passed at the state level to mitigate the interruption of medication access in times of disaster. For example, the Texas legislature passed the Texas Pharmacy Act (Sec. 562.054) and board rule (291.34), which allow a pharmacist to provide a patient with a 30-day supply of a prescription drug if a natural or manmade hazard prevents the pharmacist from contacting the practitioner. Despite these efforts, serious deficiencies in emergency preparedness for older adults continue to persist.

Recognizing the population growth of older adults calls for the need to ensure that practical, adequate, and appropriate measures are in position to deter the threats of disasters in this population. Previous research has investigated factors associated with disaster preparedness among older adults in the US, but there has been limited research considering disaster preparedness among minority older adults [5,13]. Using a local, community-focused disaster preparedness and response survey, this study aims to assess disaster preparedness in minority older adults in Houston, Texas, the fourth largest city in the U.S. and one of the most diverse. Research of this nature will provide valuable information on the needs of elderly minority communities and drive interventions to improve culturally sensitive disaster preparedness and recovery.

## 2. Methods

### 2.1. Data

Working with community-based organizations and senior living centers, an electronic survey was disseminated to older adults 55+, in the Houston metroplex, between November 2020 and January 2021. The survey, available in both English and Spanish, assessed disaster planning, response, and recovery, in the target population. To ensure representativeness from minority older adults, the study team worked with Houston area community-based organizations and senior living centers already working with African American and/or Latinx older adults. A total of 655 individuals consented to participate in the anonymous survey with overall survey completion rate of 91%. Of the complete responses, 522 represented African American and Latinx older adults. A subset of authors assessed the survey constructs for face validity.

Specifically, the disaster-preparedness probe asked: “What steps have you taken to prepare for the types of emergencies and disasters that might occur in our community?” Options included (a) Collected preparedness information, (b) Attended meetings dealing with preparedness, (c) Prepared and discussed family emergency plan, (d) Have copies of legal documentation (e.g., State-issued identification card), (e) Have document in place to prove ownership (of home, vehicle etc.), (f) Have a medical plan in place (e.g., medical needs/supplies), (g) Collected a 14-day supply of water and food, (h) Ensured you have a supply of 3-month prescription medications, (i) Ensured you have a 3-month supply of nonprescription drugs and health supplies (ex. masks, hand sanitizer, alcohol wipes, aspirin), (j) Ensured you have a battery-operated flashlight and radio, (k) Obtained physical and electronic copies of any medical records and prescriptions and (l) Have money set aside for an emergency. Respondents were asked to check as many options were appropriate. 

Responses to these 12 disaster preparedness activities informed the dependent variables for this study (see Section 2.2 measurement section). 

### 2.2. Measurement

**Figure 1:**
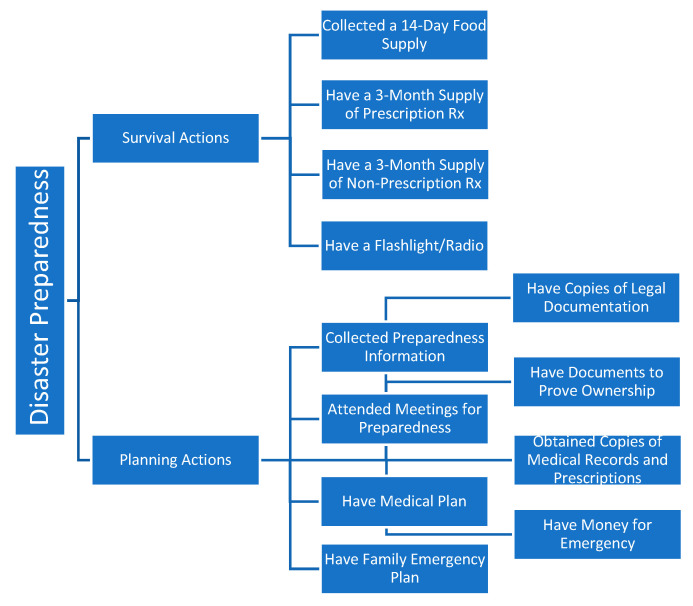
Classification of Disaster Preparedness Activities into Survival and Planning Preparedness Actions.

Based on earlier works on disaster preparedness, disaster preparedness actions are grouped into three categories: survival actions, planning actions and physical actions [14,15,16]. Survival actions include activities such as collecting a 14-day supply of water and food, having a supply of 3-month prescription medications, having a 3-month supply of nonprescription drugs and health supplies, and having a battery-operated flashlight and radio. Planning actions include activities such as collecting preparedness information, attending preparedness meeting, discussing family emergency plan, having legal plans in place, having a medical plan in place, obtaining physical and electronic copies of any medical records and prescriptions, and setting aside money for an emergency. Physical actions include activities that entail structural modifications such as fixing metal shutters to windows [14,15]. Adopting this empirical framework in a COVID-19 era, this research focused on disaster-related survival and planning actions only (physical actions that entail structural modifications are less applicable for pandemic-type disaster events and were not included in this work). Accordingly, responses to these 12 disaster preparedness activities were grouped into two distinct categories, survival actions and planning actions, as shown in Figure 1.

The dependent variables, survival actions and planning actions, were individually calculated using sum scores (used previously by Al-rousan et al., 2014). A sum score is the sum of the individual item scores within each category, so that higher scores indicate better preparedness. Aligning with earlier disaster preparedness research that used a 50% cut-off with sum scores, if more than 50% of actions in each category were completed, then the respondent was categorized as prepared [14]. Accordingly, respondents were flagged as completing survival actions if 50% or more of the survival activities were completed. Older adults who completed 50% or more of the planning activities were flagged as completing planning actions. Other covariates included sociodemographic characteristics, health insurance status, homeownership, positive COVID test in the past month (for self or close family), living arrangement (living alone, with spouse/partner, family) caregiving needs and physical limitations due to a health condition.

### 2.3. Analysis

Descriptive analyses employing frequencies and proportions were used to describe subject demographic characteristics. Chi-square tests were used to assess independent bivariate associations between respondent characteristics and (1) survival disaster preparedness actions, and (2) and planning disaster preparedness actions. A multivariate logistic regression model examined the strength of the relationships, adjusting for demographic characteristics (age, gender, race/ethnicity, marital status), socioeconomic status (education and income), positive COVID-19 test in the past month (for self or close family), living arrangement, caregiving needs and physical limitations due to a health condition. Results from the regression model were stratified by race/ethnicity to examine the correlates of survival and planning disaster preparedness actions in non-Hispanic Black/African American and Hispanic older adults, separately. This study was approved by a university institutional review board in October 2020 (IRB ID: STUDY00002584). All data management and analyses were performed using Stata 16.1. All statistical tests were 2-sided, and findings were considered statistically significant at *p* < 0.05.

## 3. Results

### 3.1. Sample Characteristics

Overall, the sample consisted of 522 older minority adults. Survey respondents comprised of 77% females, 57% Non-Hispanic Blacks, and 43% Hispanic. Thirty-three percent of the sample represented older adults 55–64 years, 42% represented older adults 65–74 years, while 25% of the sample represented adults 75 years or older. Regarding education level, 47% of survey respondents had a high school diploma or less, while 43% of respondents had an annual income less than USD 25,000. Twenty-four percent of respondents reported they had no insurance coverage, 44% had Medicare, 8% had Medicaid, 2.5% had TRICARE/Military/VA/CHAMP and 22% reported having private health insurance. 61% of survey respondents were homeowners, 72% reported unmet caregiving needs, 22% had limited activity due to a health condition, and 52% either tested positive for COVID-19 or had a close family member who tested positive for COVID-19. While 24% of respondents reported living alone, 28% lived with a partner or spouse and 48% lived with family. Overall, 42% of the sample reported being disaster prepared.

### 3.2. Bivariate Analysis

Table 1 shows the sample characteristics by disaster preparedness action categories. Taking survival actions varied by race/ethnicity (64.4% non-Hispanic Black vs. 35.6% Hispanic) as well as education level (18.4% less than high school vs. 20.8% high school diploma/GED vs. 31.1% some college/technical school vs. 16.8% Bachelor’s degree vs. 12.9% graduate degree). Disaster-related survival actions varied by home ownership (64.4% yes vs. 35.6% no) and experiencing limited activity due to a health condition (24.9% yes vs. 75.1% no). Taking survival actions also varied by income level (37.1% less than USD 25,000 vs. 41.7% of those with income between USD 25,000 and USD 74,999 vs. 21.1% of those with incomes of USD 75,000 or more) as well as insurance coverage status (17.2% uninsured vs. 6.0% Medicaid vs. 50.5% Medicare vs. 23.6% private health insurance vs. 2.7% TRICARE/Military/VA/CHAMP). Having a positive COVID-19 test (for self or close family member in the past month) was associated with taking survival actions (56.5% yes vs. 43.5% no). Taking survival actions varied by living arrangement (26.9% living alone vs. 30.4% living with a partner/spouse vs. 42.8% living with family).

Taking planning actions varied by race/ethnicity (63.2% non-Hispanic Black vs. 36.8% Hispanic), education level (19.0% less than high school vs. 22.9% high school diploma/GED vs. 28.1% some college/technical school vs. 16.9% Bachelor’s degree vs. 13.0% graduate degree), and income level (37.6% less than USD 25,000 vs. 41.3% USD 25,000–USD 74,999 vs. 21.1% USD 75,000 or more). Insurance coverage status was also significantly associated with taking planning actions (18.4% uninsured vs. 5.5% Medicaid vs. 50.6% Medicare vs. 22.6% private health insurance vs. 3.0% TRICARE/Military/VA/CHAMP). Having a positive COVID-19 test (for self or close family member) was significantly related to preparedness by taking planning actions (57.3% yes vs. 42.7% no) and taking planning actions varied by home ownership (63.5% yes vs. 36.5% no). Taking planning actions varied by living arrangement (26.6% living alone vs. 30.5% living with a partner/spouse vs. 42.9% living with family).

### 3.3. Logistic Regression Models

Table 2 shows the adjusted relationship between demographic characteristics of respondents and disaster preparedness survival and planning actions. Compared to respondents without health insurance coverage, those with Medicare had 2.54 times greater odds of taking survival actions and 3.65 times greater odds of taking planning actions. Those who had tested positive for COVID-19 (or had a close family member who had tested positive for the disease) had 2.12 greater odds of taking planning actions. 

Results of the race/ethnicity stratified model are presented in Table 3. Hispanic respondents with Medicare coverage had 3.48 times greater odds of taking survival actions and 4.52 times greater odds of taking planning actions. Compared to non-Hispanic respondents, Hispanics who had tested positive for COVID-19 themselves (or had a close family member who had tested positive for the disease) had 4.09 greater odds of taking planning actions. For non-Hispanic Black respondents, having a positive COVID-19 test increased the likelihood of taking survival actions. Compared to non-Hispanic Black respondents with an annual income less than USD 25,000, non-Hispanic Black respondents with an annual income between USD 25,000 and USD 74,999 had 4.29 greater odds of taking survival actions.

## 4. Discussion

This study assessed the correlates of disaster preparedness among community-dwelling older minority adults in Houston and explored unique differences for African American and Hispanic older adults, providing a rare glimpse into disaster preparedness among populations typically seen as more under-resourced. Houston is a particularly fitting location to study disaster preparedness of Black and Hispanic communities because these minority groups collectively comprise more than half of the city’s population. These findings are not only important for Houston but also critical for the future of our nation as Black and Hispanic populations are projected to constitute 40% of the U.S. population by 2060 [17]. As the number of Black and Hispanic Americans over the age of 65 increases as a proportion of total population, it is imperative to examine correlates of disaster-preparedness, specifically for communities experiencing successive disasters. Overall, our findings emphasize that disasters tend to expose disparities, especially racial and socioeconomic ones and older adults who had prior negative disaster experiences, higher-incomes and Medicare insurance were more likely to be prepared for a disaster. Despite a greater vulnerability to the adverse impact of disasters, we found that about 6 in 10 older adults were unprepared. These findings are significant and align with prior studies examining disaster preparedness and response, in the general older adult population. 

### 4.1. Health Insurance and Health Care Providers

Our findings suggest that having health insurance—specifically Medicare coverage—is associated with survival and planning disaster-preparedness actions. In the race/ethnicity stratified model, this finding remained significant suggesting strong associations between Medicare coverage and disaster preparedness actions, regardless of race. Importantly, this suggests that insurers may serve as an effective avenue for individuals to receive disaster preparedness information and training. Merchant et al. (2015) noted that health insurance plans have great potential in improving preparedness by a variety of strategies, including but not limited to establishing benchmarks, adjusting policies to address disasters specifically, improving communication, and creating forums for the exchange of preparedness information [18]. Considering that only Medicare coverage was protective, there is an opportunity for State Medicaid and private insurance plans to further promote disaster preparedness resources, especially to older adult beneficiaries. Placing an emphasis on preparedness corresponds to mitigating and reducing the risk of long-term health consequences that disasters may impose on these vulnerable populations [19]. For private insurance plans, disaster preparation may prevent worse health outcomes and reduce overall healthcare spending.

Medical providers that oversee health for minority older adults can also play instrumental roles in helping their patients think about wellness plans that include planning for disasters. Administrative staff in medical offices can play an important role in helping older adults create preparedness checklists and encourage them to take steps to prepare for emergencies. In addition to health care directives, medical plans should include instructions about securing legal documentation of assets, attending preparedness meetings, and accessing medical information and medication. Social workers can also be involved in assessing disaster preparedness plans, and direct older adults to resources that can increase their likelihood of success in the event of an emergency. Public health officials and insurance companies should consider incentivizing medical providers to integrate disaster preparedness activities in regions that are more prone to these emergencies.

### 4.2. Socioeconomic Stability

Stratifying by race highlights the role of income for minority populations. While income did not attain statistical significance in the overall survival or planning model, the stratified model provides evidence that low-income African American older adults who have an annual income of USD 25,000 a year are less likely to perform survival or planning disaster preparedness actions. This finding suggests that factors indicating socioeconomic stability contribute to disaster preparedness. Previous literature supports this notion as well: for example, in evaluating the impact of Hurricane Katrina, Masozera et al. (2007) found that the districts of New Orleans with greater proportions of residents living in poverty were least prepared and recovered slowest [20]. This finding supports the idea that programs that help to bolster socioeconomic security among minority older adults would subsequently have a positive impact on improved disaster preparedness as well. Garnering support for such programs may contribute to improve a variety of associated burdens, including disaster risk prevention and management.

### 4.3. The Impact of COVID-19

Our results also indicate that having tested positive for COVID-19, or having a close family member who tested positive in the past month, is associated with taking planning disaster-preparedness actions. Experiences of trauma following the COVID-19 pandemic may trigger greater preparedness for older adults who have the resources to develop these plans. The pattern of this effect slightly varies among racial minority groups. Our findings suggest that a positive COVID-19 test increases the likelihood of non-Hispanic Black individuals taking survival actions while increasing the likelihood of Hispanic individuals taking planning actions. This finding is consistent with previous research. Castañeda et al. (2020) found that direct previous disaster experience was associated with the greatest level of preparedness [21]. It is especially important to note that COVID-19-related hospitalization and deaths have disproportionately affected people of color [22]. Thus, the pandemic, which was arguably more impactful for Black and Hispanic Americans in terms of either testing positive for COVID-19 or knowing someone who tested positive, may act as motivation for minority individuals to make more of an effort to plan for disaster. Moreover, this study uniquely provides insight on disaster preparedness within racial and ethnic minority communities after the onset of the COVID-19 pandemic.

### 4.4. Cultural Sensitivity in Disaster Preparedness

It is well established that minority populations face barriers to disaster preparedness, including socioeconomic disadvantages, cultural and linguistic barriers, and distrust of government authorities [5]. Findings of a 2013 study highlight racial disparities in disaster preparedness: when compared to their non-Hispanic White counterparts, Black and Hispanic individuals were less likely to have a 3-day supply of medication [7]. Our results suggest that Hispanic individuals are more likely to be prepared for a disaster by taking survival and planning actions if they have Medicare insurance coverage. Black individuals are more likely to take survival actions with increasing income levels. Due to the differences in these two minority groups, a one-size fits all disaster preparedness program may not work.

Culturally tailored disaster preparedness meetings, in English and/or Spanish, as needed, and facilitated by the member of each respective community, are needed. In addition to providing language-specific resources, the format and delivery of information must account for unique cultural differences so that participants can relate with the content. Interventions that cater to the specific needs of minority populations are more likely to increase compliance and reap disaster preparedness benefits for communities of color. Information consistently provided by trusted voices, such as community health workers, may further cement messages that help minority older adults with disaster preparedness. Previous findings suggest that increased exposure to a greater amount of preparedness information from local health authorities is associated with preparedness [23].

This study is not without limitations. This study is focused on Houston area minority older adults, and thus, findings may not be generalizable to other large cities that do not have similar demographics. Subject responses are also subject to recall bias, but this limitation is inherent to survey studies with self-reports. Lastly, the cross-sectional nature of this study suggests that findings may differ if the survey was conducted at another point in time.

## 5. Conclusions

Since natural hazards are projected to rise in number and frequency, it is crucial to develop strategies and tools that target the needs of vulnerable minority populations. Current preparedness campaigns that focus primarily on promoting emergency kits or battery-operated flashlights are inadequate measures of preparedness for older adults, who are often limited by physical impairments, transportation options, or income for items that may be deemed non-essential to daily life. Emergency preparedness tools that not only consider immediate survival but also the importance of securing documentation needed to access assistance or rebuild lives are needed.

Older adults are already a vulnerable population during disasters and these events may be particularly disruptive to their daily living. Thus, providing survival and planning disaster preparedness resources for older minority adults is a worthwhile investment. Planning for this population necessitates innovative and culturally sensitive strategies to engage older minority adults in preparing for a potential disaster.

## Figures and Tables

**Table 1:** Sample characteristics by disaster preparedness action categories (*n* = 522).

Variables	Total (*n* = 522)		Completed * Survival Actions		Completed * Planning Actions	
	*n*	(%)	*n*	(%)	*n*	(%)
Age						
<65	174	(33.3)	128	(31.8)	124	(30.8)
65–74	219	(42.0)	165	(41.0)	175	(43.4)
≥75	129	(24.7)	109	(27.1)	104	(25.8)
Gender						
Female	394	(76.8)	299	(75.9)	299	(75.7)
Male	119	(23.2)	95	(24.1)	96	(24.3)
Race/Ethnicity						
Non-Hispanic Black	290	(56.6)	253	(64.4)	249	(63.2)
Hispanic	222	(43.4)	140	(35.6)	145	(36.8)
Marital Status						
Unmarried	266	(52.0)	214	(54.3)	213	(53.8)
Married	246	(48.1)	180	(45.7)	183	(46.2)
Education						
Less than high school	105	(21.3)	70	(18.4)	73	(19.0)
High school diploma/GED	126	(25.5)	79	(20.8)	88	(22.9)
Some college/technical school	131	(26.5)	118	(31.1)	108	(28.1)
Bachelor’s degree	77	(15.6)	64	(16.8)	65	(16.9)
Graduate degree	55	(11.1)	49	(12.9)	50	(13.0)
Income						
Less than USD 25,000	199	(43.4)	130	(37.1)	132	(37.6)
USD 25,000 to USD 74,999	173	(37.7)	146	(41.7)	145	(41.3)
USD 75,000 or more	87	(19.0)	74	(21.1)	74	(21.1)
Insurance Coverage						
Uninsured	125	(24.0)	69	(17.2)	74	(18.4)
Medicaid	40	(7.7)	24	(6.0)	22	(5.5)
Medicare	232	(44.4)	203	(50.5)	204	(50.6)
Private health insurance	112	(21.5)	95	(23.6)	91	(22.6)
TRICARE/Military/VA/CHAMP	13	(2.5)	11	(2.7)	12	(3.0)
Home Ownership						
No	206	(39.5)	143	(35.6)	147	(36.5)
Yes	316	(60.5)	259	(64.4)	256	(63.5)
Caregiving Needs						
Unmet	376	(72.0)	287	(71.4)	290	(72.0)
Met	146	(28.0)	115	(28.6)	113	(28.0)
Limited activity due to health condition						
No	358	(78.2)	265	(75.1)	264	(76.7)
Yes	100	(21.8)	88	(24.9)	80	(23.3)
Tested positive (or close family) for COVID						
No	253	(48.5)	175	(43.5)	172	(42.7)
Yes	269	(51.5)	227	(56.5)	231	(57.3)
Living Arrangement						
Living alone	125	(24.0)	108	(26.9)	107	(26.6)
Living with a partner/spouse	146	(28.0)	122	(30.4)	123	(30.5)
Living with family	251	(48.1)	172	(42.8)	173	(42.9)

% values represent proportions of respondents within columns. Percentages cannot sum horizontally because the survival and planning action categories are not mutually exclusive. * Respondents were flagged as completing survival actions if 50% or more of the survival activities were completed. Those who completed 50% or more of the planning activities were flagged as completing planning actions.

**Table 2:** Logistic Regression Modeling of the Relationship between Sociodemographic Characteristics and Survival and Planning Disaster Preparedness Actions (*n* = 522).

Variables	MV Adjusted OR	
	Survival Actions	Planning Actions
Age		
<65	Ref.	Ref.
65–74	0.75	0.95
≥75	1.16	0.78
Gender		
Female	Ref.	Ref.
Male	0.72	0.86
Race/Ethnicity		
Non-Hispanic Black	Ref.	Ref.
Hispanic	0.56	0.77
Marital Status		
Unmarried	Ref.	Ref.
Married	0.98	0.81
Education		
Less than high school	Ref.	Ref.
High school diploma/GED	0.95	0.95
Some college/technical school	2.15	0.71
Bachelor’s degree	1.27	0.74
Graduate degree	1.78	0.95
Income		
Less than USD 25,000	Ref.	Ref.
USD 25,000 to USD 74,999	1.39	1.69
USD 75,000 or more	1.19	1.46
Insurance Coverage		
Uninsured	Ref.	Ref.
Medicaid	1.14	0.89
Medicare	2.54 *	3.65 *
Private health insurance	2.10	1.65
TRICARE/Military/VA/CHAMP	1.75	4.75
Home Ownership		
No	Ref.	Ref.
Yes	1.31	1.04
Caregiving Needs		
Unmet	Ref.	Ref.
Met	1.41	1.27
Limited activity due to health condition		
No	Ref.	Ref.
Yes	1.69	0.69
Tested positive (or known someone) for COVID		
No	Ref.	Ref.
Yes	1.51	2.12 *
Living Arrangement		
Living alone	Ref.	Ref.
Living with spouse/partner	0.68	1.00
Living with family	0.90	0.83

* Signifies *p*-value < 0.05.

**Table 3:** Correlates of Survival and Planning Disaster Preparedness Actions among Hispanic and Black/African American respondents.

Variables	MV Adjusted OR			
	Survival Actions		Planning Actions	
	Hispanic (*n* = 222)	Black (*n* = 290)	Hispanic (*n* = 222)	Black (*n* = 290)
Age				
<65	Ref.	Ref.	Ref.	Ref.
65–74	0.73	0.50	0.79	1.46
≥75	0.84	3.90	0.45	1.29
Gender				
Female	Ref.	Ref.	Ref.	Ref.
Male	0.48	1.14	0.74	1.29
Marital Status				
Unmarried	Ref.	Ref.	Ref.	Ref.
Married	1.41	0.57	0.55	1.18
Education				
Less than high school	Ref.	Ref.	Ref.	Ref.
High school diploma/GED	0.84	1.98	0.56	5.14
Some college/technical school	2.40	2.82	0.83	1.50
Bachelor’s degree	1.42	2.14	0.56	2.42
Graduate degree	-	1.60	-	2.28
Income				
Less than USD 25,000	Ref.	Ref.	Ref.	Ref.
USD 25,000 to USD 74,999	0.48	4.29 *	0.88	2.39
USD 75,000 or more	1.61	2.23	6.37	1.56
Insurance Coverage				
Uninsured	Ref.	Ref.	Ref.	Ref.
Medicaid	0.86	5.77	0.76	1.44
Medicare	3.48 *	3.32	4.52 *	2.84
Private health insurance	3.94	1.96	3.17	1.22
TRICARE/Military/VA/CHAMP	1.92	1.99	-	3.63
Home Ownership				
No	Ref.	Ref.	Ref.	Ref.
Yes	1.28	1.30	0.50	2.29
Caregiving Needs				
Unmet	Ref.	Ref.	Ref.	Ref.
Met	1.17	1.53	0.90	2.03
Limited activity due to health condition				
No	Ref.	Ref.	Ref.	Ref.
Yes	2.38	1.09	0.38	0.78
Tested positive (or close family) for COVID				
No	Ref.	Ref.	Ref.	Ref.
Yes	1.09	3.03 *	4.09 *	1.44
Living Arrangement				
Living alone	Ref.	Ref.	Ref.	Ref.
Living with partner/spouse	0.68	1.12	1.77	0.87
Living with family	1.16	0.60	1.44	0.60

* Signifies *p*-value < 0.05.

## Data Availability

Per disclosure provided to study participants, we are unable to share individual level subject responses.

## References

[1] World Meterological Organization Weather-Related Disasters Increase over Past 50 Years, Causing More Damage but Fewer Deaths. https://public.wmo.int/en/media/press-release/weather-related-disasters-increase-over-past-50-years-causing-more-damage-fewer.

[2] Rajabi E., Bazyar J., Delshad V., Khankeh H.R. (2022). The Evolution of Disaster Risk Management: Historical Approach. Disaster Med. Public Health Prep..

[3] Hoffman S. (2008). Preparing for disaster: Protecting the most vulnerable in emergencies. UC Davis L. Rev..

[4] Adepoju O.E., Han D., Chae M., Smith K.L., Gilbert L., Choudhury S., Woodard L. (2021). Health Disparities and Climate Change: The Intersection of Three Disaster Events on Vulnerable Communities in Houston, Texas. Int. J. Environ. Res. Public Health.

[5] Siddiqui N.J., Purtle J.P., Andrulis D.P. (2011). Ethnicity and minority status effects on preparedness. Encyclopedia of Disaster Relief.

[6] Wolkowitz S.R. (2017). Disaster Preparedness of Independent Community-Dwelling Older Adults. Ph.D. Thesis.

[7] Bethel J.W., Burke S.C., Britt A.F. (2013). Disparity in disaster preparedness between racial/ethnic groups. Disaster Health.

[8] Williams J., Haire B. (2020). Opinion—Why Some People Don’t Want to Take a COVID-19 Test.

[9] Behr J.G., Diaz R. (2013). Disparate health implications stemming from the propensity of elderly and medically fragile populations to shelter in place during severe storm events. J. Public Health Manag. Pract..

[10] Elder K., Xirasagar S., Miller N., Bowen S.A., Glover S., Piper C. (2007). African Americans’ decisions not to evacuate New Orleans before Hurricane Katrina: A qualitative study. Am. J. Public Health.

[11] Becker J.S., Paton D., Johnston D.M., Ronan K.R., McClure J. (2017). The role of prior experience in informing and motivating earthquake preparedness. Int. J. Disaster Risk Reduct..

[12] Nepal V., Scott D., Banerjee D., Perry M. (2009). Understanding Disaster Preparedness of Linguistically Isolated Population Groups: Chinese, Vietnamese, Somali and Spanish Speaking. Community Health Statistics.

[13] Chae M., Choudhury S., Franco-Castano J., Adepoju O.E. (2022). Self-perceived disaster preparedness in minority older adults: A cross-sectional study. Am. J. Disaster Med..

[14] Al-Rousan T.M., Rubenstein L.M., Wallace R.B. (2014). Preparedness for natural disasters among older US adults: A nationwide survey. Am. J. Public Health.

[15] Prior T., Eriksen C., Paton D., Tedim F. (2012). What does being ‘well prepared’ for wildfire mean. Wildfire and Community: Facilitating Preparedness and Resilience.

[16] Russell L.A., Goltz J.D., Bourque L.B. (1995). Preparedness and hazard mitigation actions before and after two earthquakes. Environ. Behav..

[17] Vespa J., Armstrong D.M., Medina L. (2018). Demographic Turning Points for the United States: Population Projections for 2020 to 2060.

[18] Merchant R.M., Finne K., Lardy B., Veselovskiy G., Korba C., Margolis G.S., Lurie N. (2015). State of emergency preparedness for US health insurance plans. Am. J. Manag. Care.

[19] Toner E.S., McGinty M., Schoch-Spana M., Rose D.A., Watson M., Echols E., Carbone E.G. (2017). A community checklist for health sector resilience informed by Hurricane Sandy. Health Secur..

[20] Masozera M., Bailey M., Kerchner C. (2007). Distribution of impacts of natural disasters across income groups: A case study of New Orleans. Ecol. Econ..

[21] Castañeda J.V., Bronfman N.C., Cisternas P.C., Repetto P.B. (2020). Understanding the culture of natural disaster preparedness: Exploring the effect of experience and sociodemographic predictors. Nat. Hazards.

[22] Lopez L., Hart L.H., Katz M.H. (2021). Racial and ethnic health disparities related to COVID-19. JAMA.

[23] Thomas T.N., Leander-Griffith M., Harp V., Cioffi J.P. (2015). Influences of preparedness knowledge and beliefs on household disaster preparedness. Morb. Mortal. Wkly. Rep..

